# Editorial: Signal Transduction in Stomatal Guard Cells

**DOI:** 10.3389/fpls.2017.00114

**Published:** 2017-02-07

**Authors:** Agepati S. Raghavendra, Yoshiyuki Murata

**Affiliations:** ^1^School of Life Sciences, University of HyderabadHyderabad, India; ^2^Graduate School of Environmental and Life Science, Okayama UniversityOkayama, Japan

**Keywords:** abscisic acid, cytosolic calcium, cytosolic pH, ion-channels, microbial elicitors, NO, ROS, secondary messengers

## Introduction

During adaptation of plants to water stress/drought, the tiny pores on the leaf surface, called “stomata,” play a very important role. Stomatal movements can modulate the entry/exit of not only CO_2_/water (Lawson and Blatt, 2014) but also microbial pathogens (Agurla et al., 2014; Arnaud and Hwang, 2015). The stomatal opening/closure is brought out by changes in the turgor of guard cells. The abiotic/biotic stress factors induce a series of changes in the signaling components of guard cells, such as ROS, NO, pH and calcium, leading to efflux of ions, loss of turgor and stomatal closure. Due to their dynamic responses to signals, and the ease of handling leaf epidermis, the stomatal guard cells have been popular systems to study signal transduction in plants.

The guard cells are extremely efficient in their signal integration to optimize stomatal aperture. Murata et al. (2015) summarized the studies on signal transduction pathway in guard cells, with emphasis on downstream components. Extensive work has been carried out using the plant hormones, such as abscisic acid (ABA) and methyl jasmonate (Assmann and Jegla, 2016). Similarly, the elicitors, such as chitosan and flagellin, are also used to study sensing and transduction of signals (Agurla et al., 2014). Guard cells are unique in not only their ability to respond to external signals but also their structure and development. Very few groups are working on development and differentiation of guard cells (Chater et al., 2014; Keerthisinghe et al., 2015; Torii, 2015).

Besides the areas covered in the present research topic, there are additional aspects of contemporary interest. Some of these are: signaling by plant lipids in relation to guard cell function (Puli et al., 2016), molecular mechanisms of sensing CO_2_ (Engineer et al., 2016), signals from underlying mesophyll cells of leaf (Lawson et al., 2014) and cross-talk of ABA with ethylene and brassinosteroids during stomatal closure (Shi et al., 2015). Another area is the systems biology to integrate and model the signaling network in guard cells (Medeiros et al., 2015).

## Articles in the research topic

There have been several reviews on signaling components during stomatal closure, which are in different journals. The present research topic has been planned to provide a set of articles as a compendium and a ready source of information for all those interested in guard cell function.

Most of the work on signal transduction in guard cells has been with ABA and MJ, while such studies with microbial elicitors are limited. The guard cells perceive the presence of microbes though the microbe associated molecular patterns (MAMPs). The signaling events initiated by MAMPs overlap with the effects of ABA, particularly with reference to the rise in ROS, NO, cytosolic Ca^2+^ and activation of ion channels (Ye and Murata). Agurla and Raghavendra assessed the multiple signaling components induced by plant hormones or microbial elicitors. They proposed that reactive oxygen species (ROS), cytosolic free Ca^2+^ and ion channels are the major converging points while ROS, NO and cytosolic free Ca^2+^ are points of divergence. The end result is the ion channel modulation causing an efflux of K^+^/Cl^−^/malate from guard cells leading to stomatal closure. The major role of ROS and NO in guard cells during the stomatal closure is well established (Gayatri et al., 2013; Song et al., 2014). However, the role of NO is quite intriguing as NO can either amplify or limit (by scavenging) the effects of ROS (Laxalt et al.). Further, other gasotransmitters such as H_2_S can also regulate stomatal aperture (Scuffi et al.).

Abscisic acid induces not only stomatal closure, but also integrates multiple physiological processes, including leaf senescence. Using mutants, Song et al., describe how ABA can regulate the components of senescence, namely gene expression, calcium channel activation in plasma membrane, loss of chlorophyll and ion leakage. Thus, ABA action through Ca^2+^ signaling appears to function during leaf senescence as well.

Protein phosphorylation is an important strategy for integrating different signals in guard cells (Zhang et al., 2014; Vilela et al., 2015). Often the signal transduction processes involve mitogen-activated protein kinases (MAPK), which and drive the cascade of events. Lee et al. highlight the advances in the MAPK-mediated guard cell signaling. These kinases mediate phosphorylation of their next target protein. Balmant et al. describe the methods to study post translational modification (PTM) and redox modification of guard cell proteins. With improved technology, further studies on PTM are bound to intensify and reveal interesting insights. For example, reactive carbonyl species function downstream of ROS production in abscisic acid signaling in guard cells (Islam et al., 2016). Similarly, the 14-3-3 proteins could target and modify different proteins in guard cells (Cotelle and Leonhardt).

The role of guard cell sugars in the stomatal movement is acknowledged, but detailed studies are lacking. Using citrus plants with over-expressed hexokinase I in the guard cells, Lugassi et al. provide a convincing study that hexokinase regulates photosynthesis and promotes stomatal closure in not only annual species, but also in perennials. The description of an optimized procedure for the isolation of abaxial epidermal peels from grasses, including barley, wheat and *Brachypodium*, to study their responses to ABA and CO_2_ (Shen et al.), would open up an exciting range of possibilities.

## Concluding remarks

The articles in our research topic provide interesting leads for future work. The stomatal guard cells are excellent models to study PTM of proteins by ROS as well as NO during signal transduction. Such PTM studies could explain the interactions of 14-3-3 proteins with MAP kinases in guard cells. Hexoses can contribute to the guard cell osmoticum, but their origin from within guard cells or mesophyll cells needs to be investigated. A rise in ROS, NO and cytosolic pH of guard cells is essential for stomatal closure, but their exact sequence and their interactions are quite interesting for further studies. The signaling events initiated by MAMPs are fairly understood, but the identity of MAMP-receptors is to be established.

## Author contributions

AR and YM assessed the information in the Frontiers articles, as well as the available literature. Both AR and YM drafted and finalized the manuscript together.

### Conflict of interest statement

The authors declare that the research was conducted in the absence of any commercial or financial relationships that could be construed as a potential conflict of interest.
